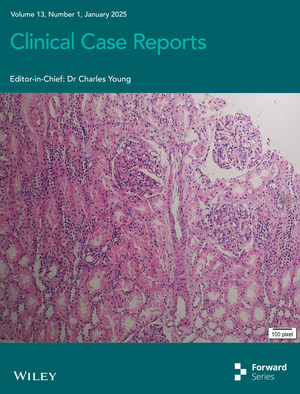# Cover Image

**DOI:** 10.1002/ccr3.70133

**Published:** 2025-01-16

**Authors:** Ayman Azhary, Mohammed Taha, Nooh Mohamed Hajhamed, Salahaldeen Ismail Mohammed, Nouh Saad Mohamed, Waleed Azhary Sir Alkhatim

## Abstract

The cover image is based on the article *A Case Report of Membranoproliferative Glomerulonephritis: Infection‐Related or Immune‐Related?* by Ayman Azhary et al., https://doi.org/10.1002/ccr3.70088.